# Supplementary figures and images for: Branched actin polymerization drives invasive protrusion formation to promote myoblast fusion during mouse skeletal muscle regeneration
Source: eLife. 2026 Jan 29;14:RP103550. doi: 10.7554/eLife.103550 (PMC12854672; doi:10.7554/eLife.103550)

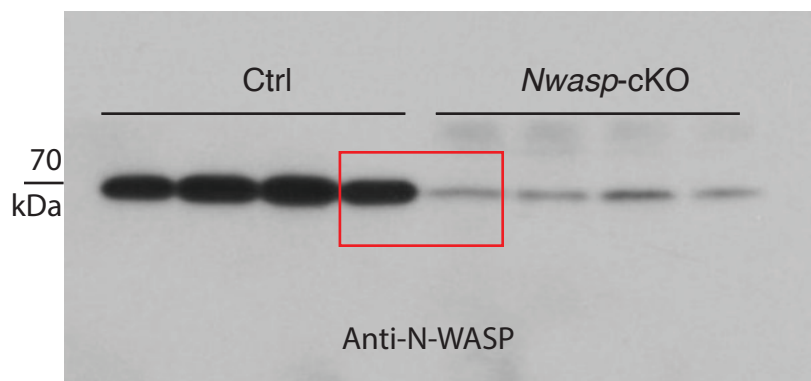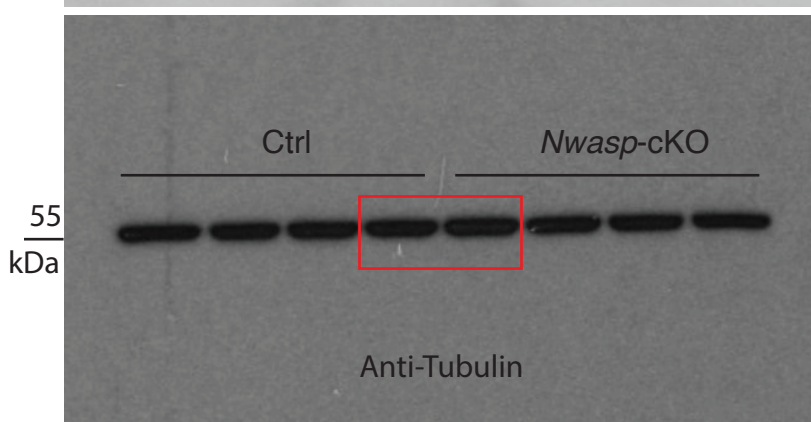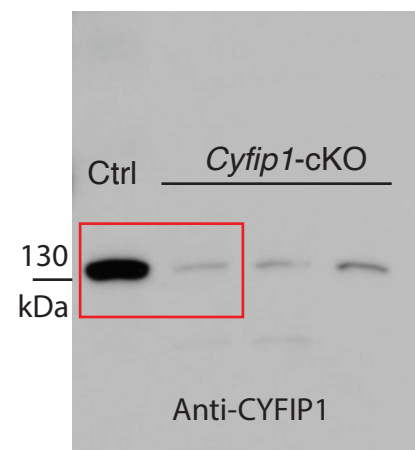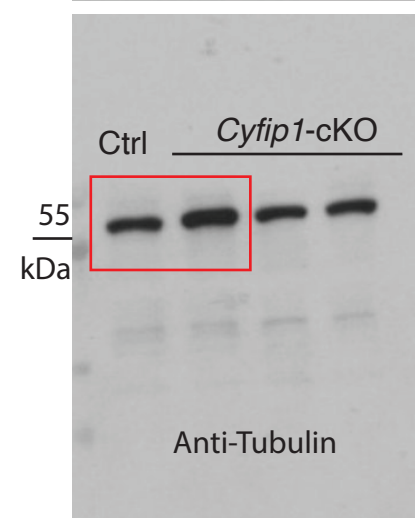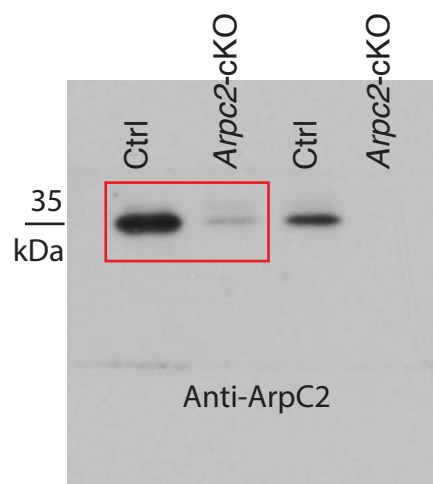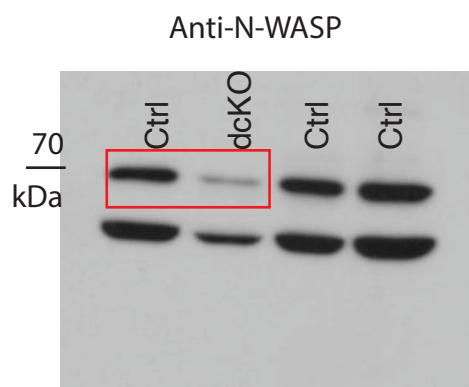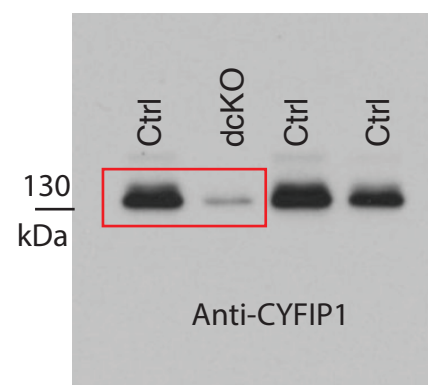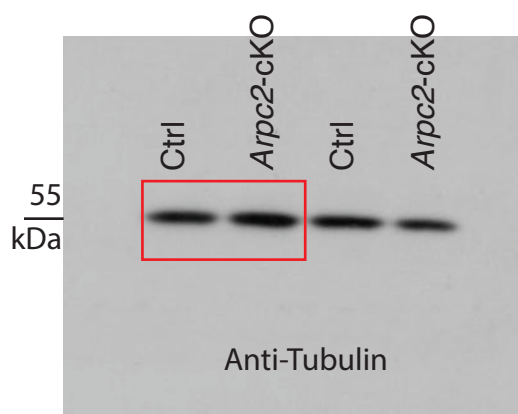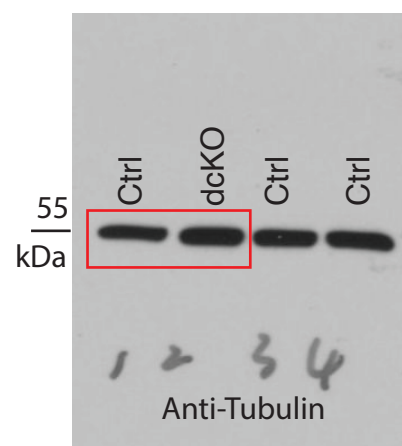

Supplement: Figure 2—figure supplement 1—source data 1. [file elife-103550-fig2-figsupp1-data1.pdf]

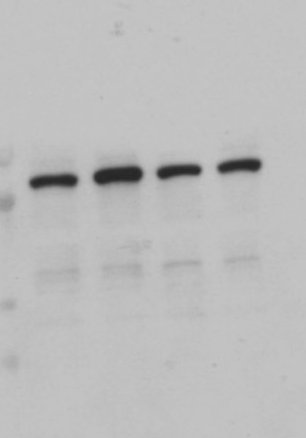

Supplement: Figure 2—figure supplement 1—source data 2. [file elife-103550-fig2-figsupp1-data2.zip › Figure 2-figure supplement 2-source data 1/Cyfip1 cKO_anti-Tubulin.jpg]

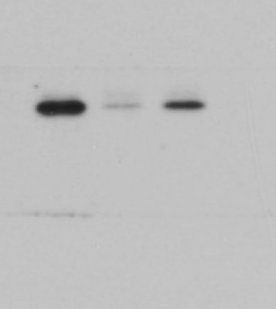

Supplement: Figure 2—figure supplement 1—source data 2. [file elife-103550-fig2-figsupp1-data2.zip › Figure 2-figure supplement 2-source data 1/Arpc2-cKO_anti-ArpC2.jpg]

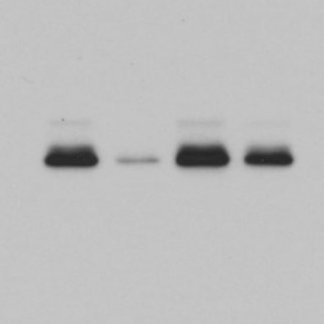

Supplement: Figure 2—figure supplement 1—source data 2. [file elife-103550-fig2-figsupp1-data2.zip › Figure 2-figure supplement 2-source data 1/dcKO_anti-CYFIP1.jpg]

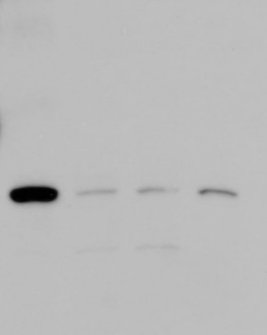

Supplement: Figure 2—figure supplement 1—source data 2. [file elife-103550-fig2-figsupp1-data2.zip › Figure 2-figure supplement 2-source data 1/Cyfip1-cKO_anti-CYFIP1.jpg]

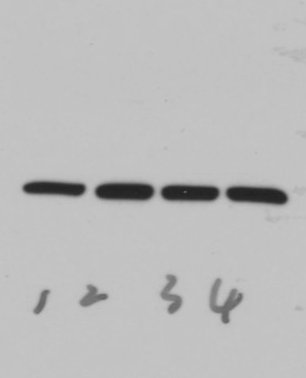

Supplement: Figure 2—figure supplement 1—source data 2. [file elife-103550-fig2-figsupp1-data2.zip › Figure 2-figure supplement 2-source data 1/dcKO_anti-Tubulin.jpg]

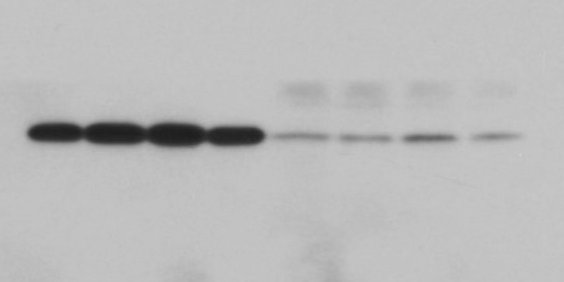

Supplement: Figure 2—figure supplement 1—source data 2. [file elife-103550-fig2-figsupp1-data2.zip › Figure 2-figure supplement 2-source data 1/Nwasp-cKO_anti-N-WASP.jpg]

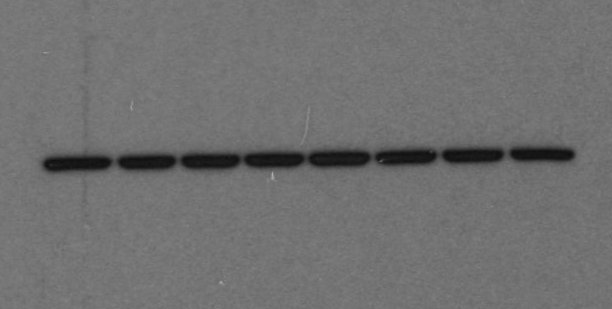

Supplement: Figure 2—figure supplement 1—source data 2. [file elife-103550-fig2-figsupp1-data2.zip › Figure 2-figure supplement 2-source data 1/Nwasp-cKO_anti-Tubulin.jpg]

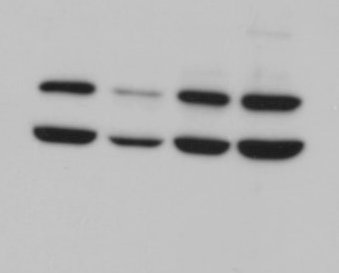

Supplement: Figure 2—figure supplement 1—source data 2. [file elife-103550-fig2-figsupp1-data2.zip › Figure 2-figure supplement 2-source data 1/dcKO_anti-N-WASP.jpg]

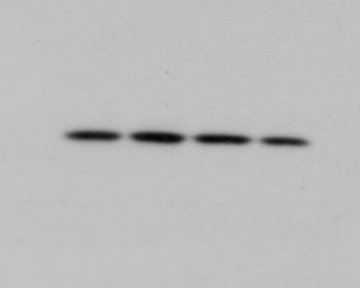

Supplement: Figure 2—figure supplement 1—source data 2. [file elife-103550-fig2-figsupp1-data2.zip › Figure 2-figure supplement 2-source data 1/Arpc2-cKO_anti-Tubulin.jpg]

Anti-MymX

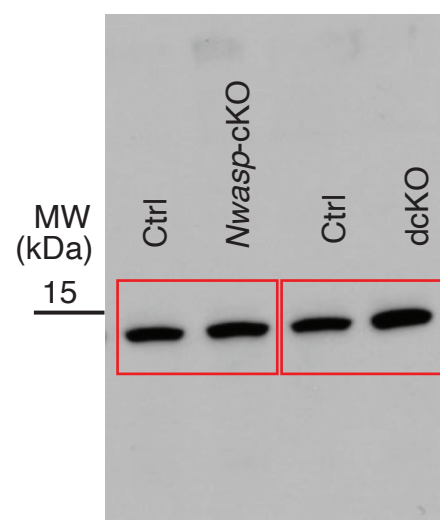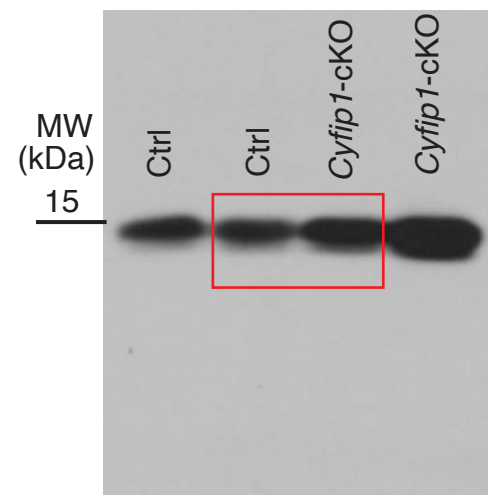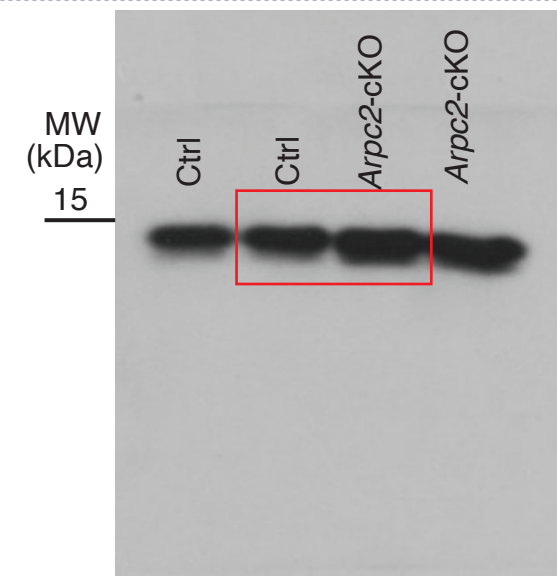

Anti-MymK

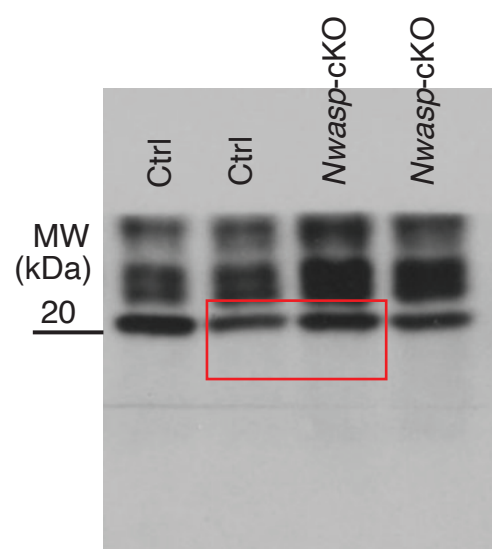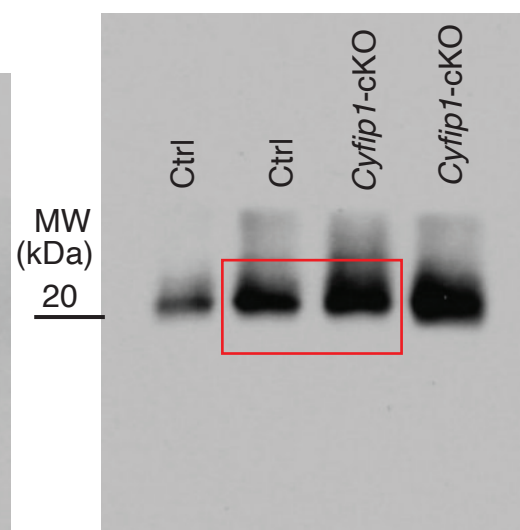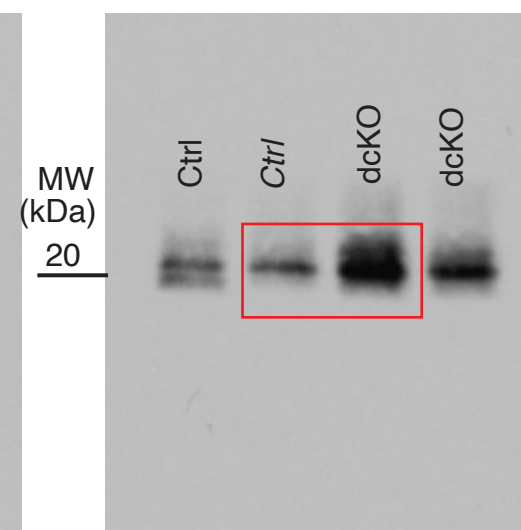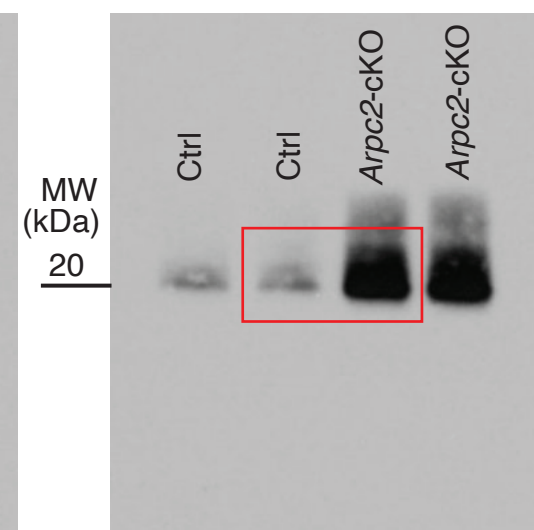

Anti-Tubulin

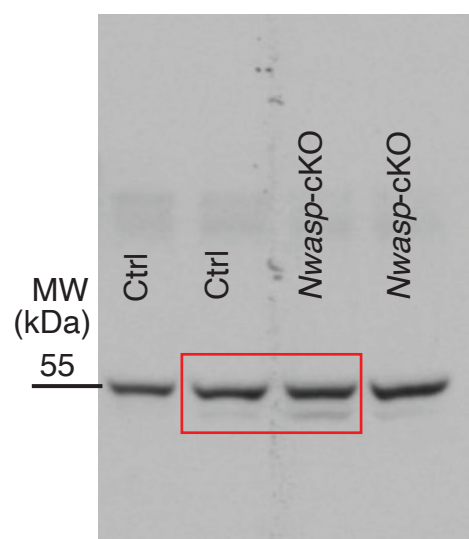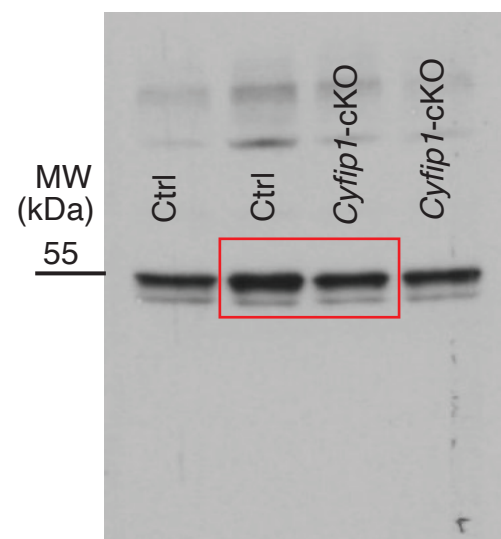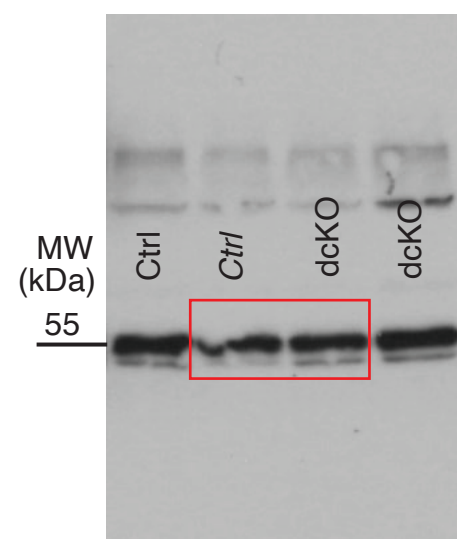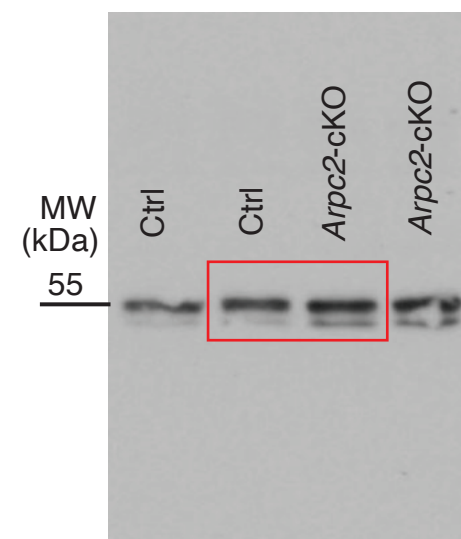

Supplement: Figure 3—figure supplement 1—source data 1. [file elife-103550-fig3-figsupp1-data1.pdf]

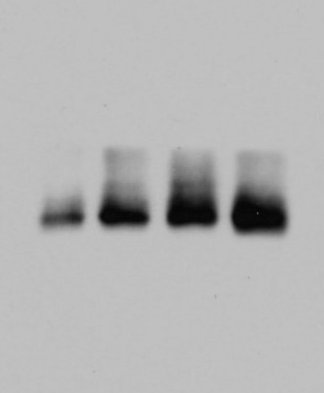

Supplement: Figure 3—figure supplement 1—source data 2. [file elife-103550-fig3-figsupp1-data2.zip › Figure 3-figure supplement 1-source data 1/Cyfip1-cKO_anti-MymK.jpg]

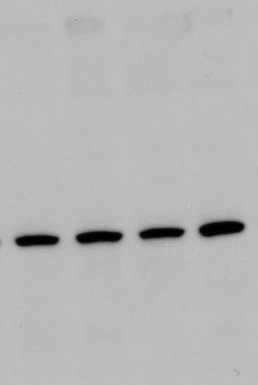

Supplement: Figure 3—figure supplement 1—source data 2. [file elife-103550-fig3-figsupp1-data2.zip › Figure 3-figure supplement 1-source data 1/Nwasp and dcKO_anti-MymX.jpg]

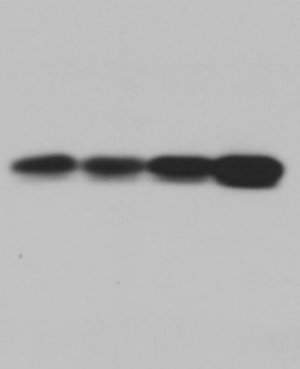

Supplement: Figure 3—figure supplement 1—source data 2. [file elife-103550-fig3-figsupp1-data2.zip › Figure 3-figure supplement 1-source data 1/Cyfip1-cKO_anti-MymX.jpg]

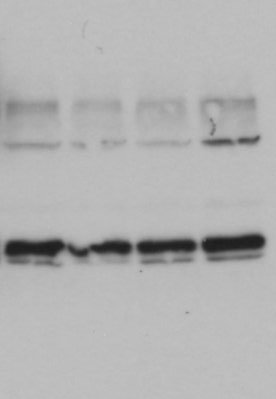

Supplement: Figure 3—figure supplement 1—source data 2. [file elife-103550-fig3-figsupp1-data2.zip › Figure 3-figure supplement 1-source data 1/dcKO_anti-Tubulin.jpg]

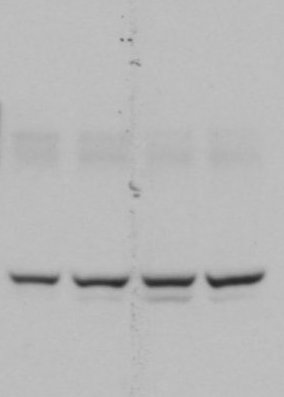

Supplement: Figure 3—figure supplement 1—source data 2. [file elife-103550-fig3-figsupp1-data2.zip › Figure 3-figure supplement 1-source data 1/Nwasp-cKO_anti-Tubulin.jpg]

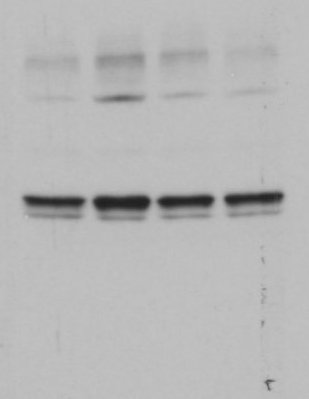

Supplement: Figure 3—figure supplement 1—source data 2. [file elife-103550-fig3-figsupp1-data2.zip › Figure 3-figure supplement 1-source data 1/Cyfip1-cKO_anti-Tubulin.jpg]

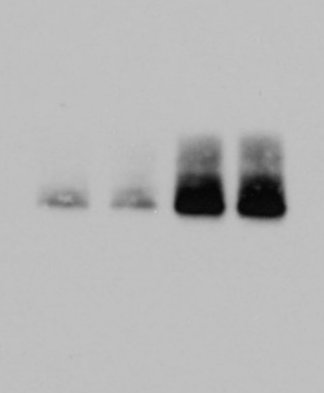

Supplement: Figure 3—figure supplement 1—source data 2. [file elife-103550-fig3-figsupp1-data2.zip › Figure 3-figure supplement 1-source data 1/Arpc2-cKO_anti-MymK.jpg]

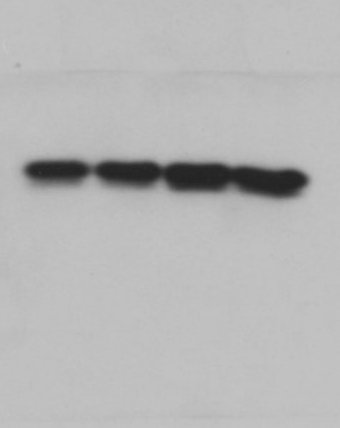

Supplement: Figure 3—figure supplement 1—source data 2. [file elife-103550-fig3-figsupp1-data2.zip › Figure 3-figure supplement 1-source data 1/Arpc2-cKO_anti-MymX.jpg]

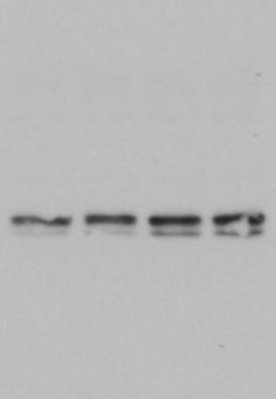

Supplement: Figure 3—figure supplement 1—source data 2. [file elife-103550-fig3-figsupp1-data2.zip › Figure 3-figure supplement 1-source data 1/Arpc2-cKO_anti-Tubulin.jpg]

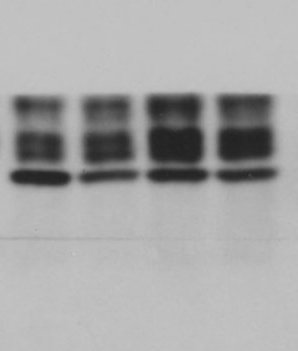

Supplement: Figure 3—figure supplement 1—source data 2. [file elife-103550-fig3-figsupp1-data2.zip › Figure 3-figure supplement 1-source data 1/Nwasp-cKO_anti-MymK.jpg]

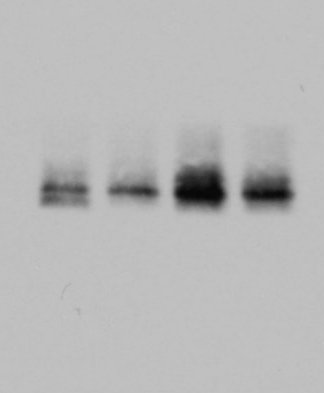

Supplement: Figure 3—figure supplement 1—source data 2. [file elife-103550-fig3-figsupp1-data2.zip › Figure 3-figure supplement 1-source data 1/dcKO_anti-MymK.jpg]
